# Mineralising and antibacterial effects of modified calcium phosphate treatment on human root cementum

**DOI:** 10.1186/s12903-016-0246-4

**Published:** 2016-07-19

**Authors:** Haijing Gu, Junqi Ling, Xiaoyan Zhou, Limin Liu, Ziming Zhao, Jin-Long Gao

**Affiliations:** Guanghua School of Stomatology, Hospital of Stomatology, Sun Yat-sen University & Guangdong Provincial Key Laboratory of Stomatology, 56 Ling Yuan Xi Road, Guangzhou, 510055 China; Department of Biomaterials & Biomimetics, New York University College of Dentistry, New York City, USA; Faculty of Dentistry, University of Sydney, Sydney, NSW Australia; Institute of Dental Research, Westmead Centre for Oral Health, Westmead Millennium Institute, Westmead, 2153 NSW Australia; Guangdong Provincial Institute of Traditional Chinese Medicine, Guangzhou, China

**Keywords:** Root caries, Cementum, Modified calcium phosphate, Remineralisation, Anti-microbial

## Abstract

**Background:**

Aging population will lead to the increase of incidence of root caries globally. The clinical management of root caries is challenging due to the difficulty in moisture isolation. The root caries is caused by the release of organic acids from cariogenic bacteria which results in the dissolution of cementum and dentin of the root. The purpose of this study is to study the efficacy of modified saturated calcium phosphate solution (CaP) supplement with zinc (Zn^2+^) and/or fluoride (F^-^) in providing root cementum surfaces less susceptible to acid dissolution and bacterial colonization.

**Methods:**

Human root cementum sections from extracted premolars were treated with three modified calcium phosphate solutions (M/A-CaPs) respectively: (A) CaP-F/Zn, supplemented with F^-^ and Zn^2+^; (B) CaP-F, supplemented with F^-^ only; (C) CaP-Zn, supplemented with Zn^2+^ only. The surface characteristics of treated cementum sections were investigated using scanning electron microscopy (SEM) and fourier transform infrared spectroscopy (FT-IR). Following the acid attack and *Streptococcus mutans* challenge, M/A-CaPs treated cementum surfaces were analysed using inductive coupled plasma (ICP) and SEM respectively.

**Results:**

Compared with the control group, M/A-CaPs treated cementum presented significant improvements in resistance to acid dissolution and bacterial colonization. Among M/A-CaPs, the CaP-F/Zn treated cementum surfaces released the lowest amount of Ca^2+^ ions (2.11 ± 0.51 ppm) upon acid challenge (*n* = 3, *p < 0.01*) and also presented the most significant inhibiting effect against the colonization of *S. mutans* (*n* = 180, *p < 0.*05)*.*

**Conclusions:**

Saturated calcium phosphate solution CaP supplemented with both F^-^ and Zn^2+^ could be applied as an effective coating material providing acid resistance and antibacterial property on cementum surfaces. The modified calcium phosphate-based solution could be a new treatment strategy to prevent the development of root caries and arrest the further progression of root caries.

## Background

Owing to the growing population of elderly people worldwide, root caries is becoming an urgent issue in geriatric dentistry [1, 2]. As one of the major causes of tooth loss in elderly, the prevalence of root caries has been revealed nearly half in the aging population by the recent studies [3, 4]. Du M et al. reported that the root surface caries prevalence rates were 13.1 % in the middle-aged group and 43.9 % in the elderly group. Prevalence increased with aging, such that by age 75 and over, over 50 % had one or more root surface lesions [5]. Compared with coronal caries, the clinical management of root caries is more challenging with respect to the limited accessibility and difficulty in tooth isolation.

Root caries differs from coronal caries primarily due to different tissue compositions forming the outer layer of the root. Coronal caries primarily begins in enamel, a highly mineralised tooth structure with 96 % mineral. Root caries, however, involves the less mineralised tissue, cementum, which contains only 50 % mineral. The natural root surface is covered by a cementum layer of varying thickness. Similar to enamel, the cementum could function as a barrier against the diffusion of the mineral ions out of the lesion and provide initial caries resistance for the root surface [6]. However, the cementum is a thinner layer of less mineralised tissue with considerably varied thickness, especially the cervical third with only 16–60 μm thickness. It is susceptible to routine oral prophylaxis and periodontal treatments such as scaling and root planning where the cementum can be easily disintegrated or removed at the cementoenamel junction level even down to the coronal third of the root. Due to traumatic tooth brushing, periodontal diseases [7, 8], bleaching, and orthodontic movement of teeth [9], gingival recessions were found in more than 60 % of the younger population (<20 years) and more than 90 % of the older population (>50 years). It causes the early exposure of cementum on the root surface which increases susceptibility to root caries [10, 11].

The clinical features and locations of root caries cause technical difficulties in early diagnosis and treatment. Hence, more efforts should be made on the prevention and early management of this disease. Among the mineralised tissues, fluoride concentration of cementum is the greatest and it increases with age [12, 13] or with F^-^ exposure [14, 15]. Intact cementum layer has intrinsic ability to protect the underlying dentin against acidic demineralisation via ions uptakes from the surroundings and accumulating fluoride [16]. However, fluoride is not generally bactericidal which cannot effectively inhibit the growth of many cariogenic pathogens such as *lactobacilli*, *actinomyces spp*, and *streptococci* [17]. Our previous results have shown the successful colonization of *Streptococcus mutans* on the dentin surfaces treated with experimental mineralising solution containing F^-^ or F^-^ bound dentin surfaces [18].

In our previous studies, modified calcium phosphate solution (M/A-CaPs) containing both F^-^ and Zn^2+^ was found to be effective in the mineralisation of dentin surfaces, occluding dentin tubules [19], and providing antibacterial property especially at acidic pH condition [18]. The presence of F^-^ alone increased the mineralising efficiency of CaP solution and inhibited dentin dissolution by the formation of fluoridated hydroxyapatite. Zinc salt has been demonstrated to provide antibacterial property, inhibit plaque formation, and prevent gingival inflammation [20–22]. To overcome the limited antimicrobial activity of fluoride, in addition to F^-^, Zn^2+^ element was also supplemented in our experimental CaP solution. Till now, there are limited options of dentifrices and agents can effectively bind to the cementum, to form a coating layer which is resistant to acidic dissolution and bacterial colonization [23]. In this study, we compared the coating efficacy of M/A-CaPs with or without F^-^ and Zn^2+^ on the cementum remineralisation. This study would provide a new strategy to prevent the initiation of root caries especially in the susceptible population groups such as elderly people and patients suffering from gingival recession.

## Methods

### Preparation of cementum samples

The sound human premolars extracted for orthodontics reasons were collected from Guanghua School of Stomatology Sun Yan-sen University (age range, from 16 to 22), and patients suffering from periodontal diseases were not considered. After extraction, teeth were immediately stored in saline, followed by being sterilized by gamma radiation, and stored in saline (The study design, sampling method and written consent forms were approved by The Human Research Ethics Committee of Guanghua School of Stomatology, Sun Yat-sen University. All participants provided the consents before their participation in this project.). Periodontal ligament fibers were removed carefully from root surfaces under dissecting microscope with a sharp Gracey curette. Root surficial cementum was kept intact [24–26].

Following debridement and brushing with fluoride-free prophylactic paste, the crowns and lower half roots were removed using a water-cooled diamond-bladed saw (Series 15 HC Diamond, N 11-4244, Buehler, USA), to prepare upper root sections with dimensions of 6 mm × 6 mm × 2 mm. After sonication for 10 min to remove the polishing abrasive particles, the specimens were rinsed with double distilled water (DDW) and dried with compressed air.

### Preparation of M/A-CaP solutions

Calcium deficient apatites (SH874) was prepared by mixing 10 mM calcium hydroxide (Ca(OH)_2_) and 6 mM anhydrous monobasic sodium phosphate (NaH_2_PO_4_) in 100 ml DDW at 90 °C, 2 h, according to the reaction below:

10 Ca(OH)_2_ + 6NaH_2_PO_4_ → (Ca,Na)_10_(PO_4_,HPO_4_)_6_(OH)_2_

The precipitate was filtered and collected, washed with DDW three times, dried in the oven at 70 °C and characterized using X-ray diffraction (Philips X’ pert X-ray diffractometer). The M/A-CaP solutions were prepared from mixtures of SH874, NaF, and/or ZnCl_2_ as detailed in Table 1 (patent application submitted).

**Table 1:** Ingredients of each 100 ml M/A-CaP solutions

Solutions	SH_874_ (mM)	NaF (mM)	ZnCl_2_ (mM)	4.25 % H_3_PO_4_ (ml)
CaP-F/Zn	10	2	2	10
CaP-F	10	2	0	10
CaP-Zn	10	0	2	10

Solutions A (CaP-F/Zn), B (CaP-F), and C (CaP-Zn) were adjusted to pH 5.5 using sodium hydroxide. The Ca^2+^, PO_4_^3-^, and Zn^2+^ ion concentrations of the filtrates were determined by inductive coupled plasma (ICP, Thermo Jarrell Ash Model-Trace Scan Inductive Coupled Plasma, Waltham, MA) and the F^-^ ion concentration was measured by fluoride tracer (Orion, 940900).

### Treatment of the cementum samples

Sections were randomly distributed into treated groups and control group. The cementum sections were immersed with shaking (60/min) in CaP-F/Zn, CaP-F, CaP-Zn solutions or DDW for 4 min, then rinsed in DDW and dried with compressed air.

### Determination of physiochemical properties

The coatings on cementum surfaces were characterized using scanning electron microscopy (SEM) (JEOL JSM-5400; JEOL USA, Inc., Peabody, MA; and Hitachi S-3500 N; Hitachi, Ltd., Tokyo, Japan) and fourier transform infrared spectroscopy (FT-IR) (Nicolet 550; France). For the SEM analysis, the cementum sections were mounted onto aluminium stubs and sputter coated with gold. SEM images were taken under the same magnification and working distance. Of six sections in each group, ten images were selected randomly on each section and six of these SEM images were captured. This experiment was independently repeated three times. For the FT-IR analysis, the apatite powder pellet was prepared by mixing 1 mg of the powdered material scraped from the treated cementum surfaces with 250 mg KBr (IR grade) and pressing at 10,000 psi using a hydraulic press (Carver laboratory press, mode C, Ser.No.33000-577, Fred S. Carver INC). The FT-IR scan covered the range from 4000 cm^-1^ to 400 cm^-1^. Assignment of absorption bands were determined according to earlier study on carbonate apatites and standard calcium phosphates [27].

Dissolution of the coating was determined by monitoring the release of calcium ions from M/A-CaPs pre-treated cementum surfaces in weak acidic buffer (0.1 M KAc, pH 6, 37 °C) over time using ICP. This experiment was carried out after applying nail varnish on all parts of cementum sections except a circular area (diameter, 5 mm).

### Determination of anti-bacterial property

*Streptococcus mutans* strain ATCC 25175 was used to evaluate the effect of the coating on bacterial colonization on cementum surface. The bacteria from a brain heart infusion agar plate were inoculated into 200 ml brain heart infusion broth (OXOID) and incubated at 37 °C overnight. The M/A-CaPs pre-treated cementum sections were divided into 4 groups according to the treatment (18 sections/group) and put into 24-well plates. The bacterial cells were collected and adjusted to OD_600_ of 0.30 and 2 ml of bacterial culture for each well were applied to challenge the cementum. The specimens were then incubated at 37 °C in an anaerobic atmosphere. In order to ensure the specimens to be challenged by bacteria from the same growth phase, the culture was changed every 2 h. Every three cementum sections in each group were then taken out from wells at 6, 12, and 24 h and washed with phosphate-buffered saline (PBS) to remove the unbound bacterial cells.

Cementum samples were fixed in Trump’s fixative (VWR, Inc.) for 1 h and then rinsed with PBS, post-fixed for 1 h in 2 % Osmium Tetroxide (OsO_4_), rinsed with PBS then dehydrated in ethanol, and dried in vacuum desiccators. The specimens were viewed and six images of each sample were randomly captured by SEM under the 2000 × magnification. A Bioquant Nova Advanced Image Analysis apparatus (200 R&M Biometrics, Inc.) was used to count the number of bacteria on cementum surface.

### Statistical analysis

All data were assessed by SPSS15.0 using one-way analysis of variance (ANOVA) followed by Student-Newman-Keuls or Dunnett’s T3 post hoc with α =0.01. If no significant difference was obtained, data were analysed again with α =0.05.

## Results

### Solution composition

Chemical analyses showed three solutions maintaining relatively constant calcium and phosphorus levels. The fluoride concentration is constant between CaP-F/Zn and CaP-F. Similarly, the zinc concentration is constant between CaP-F/Zn and CaP-Zn (Table 2).

**Table 2:** Chemical compositions (in ppm) of the 3 solutions with relatively constant Ca and P but varying F and Zn concentrations

Solutions	F (ppm)	Ca (ppm)	P (ppm)	Zn (ppm)
CaP-F/Zn	27.21 ± 2.6	48.6 ± 5.4	102.5 ± 3.3	14.1 ± 0.5
CaP-F	27.41 ± 1.8	50.6 ± 7.3	110.8 ± 2.7	0 ^*^
CaP-Zn	0 ^*^	52.6 ± 3.1	105.0 ± 6.1	15.2 ± 0.7

Element concentrations (F, Ca, P, Zn) were compared among solutions. * *p* < 0.05, *n* = 3

### Physicochemical properties of treated cementum surfaces

**Fig. 1:**
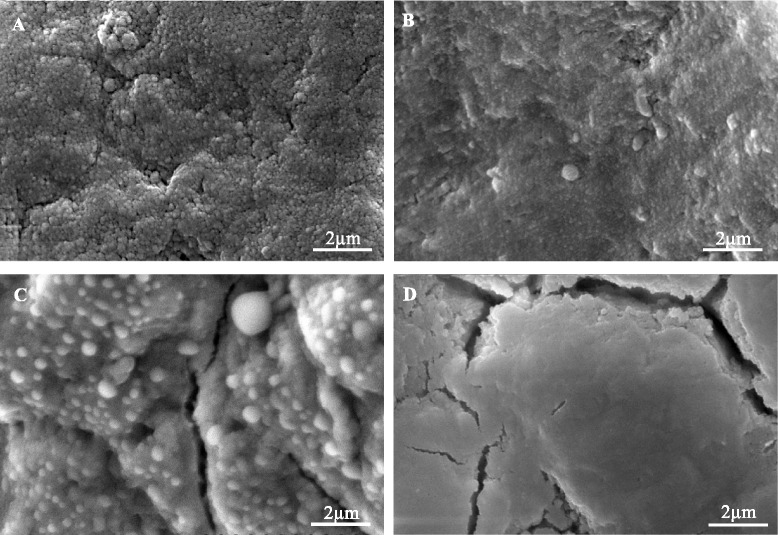
Representative SEM images of cementum: (**a**) CaP-F/Zn; (**b**) CaP-F; (**c**) CaP-Zn; (**d**) DDW. Cementum treated with CaP-F/Zn showed the greatest amount of crystal deposition followed by groups treated with either CaP-F or CaP-Zn. No crystal deposition was observed on DDW treated cementum

As shown in Fig. 1, the cementum sections treated with M/A-CaP solutions presented different amounts and various morphologies of crystal deposits on the surfaces. Cementum surfaces treated with CaP-F/Zn (Fig. 1a) and CaP-F (Fig. 1b) were shown to be coated by compact and homogenous fine crystal precipitates. In contrast, a loose crystal layer with heterogeneous precipitates was observed on the cementum surfaces treated with CaP-Zn (Fig. 1c). Notably, most of cementum cracks were filled effectively by crystal precipitates formed in CaP-F/Zn and CaP-F groups, although large cracks still could be visualised under low magnification (data not shown). The amount of crystals deposited on cementum surfaces appeared to be dependent on the composition of mineralising solutions. No crystal deposition was observed on cementum surfaces of negative control group treated with DDW (Fig. 1d).

**Fig. 2:**
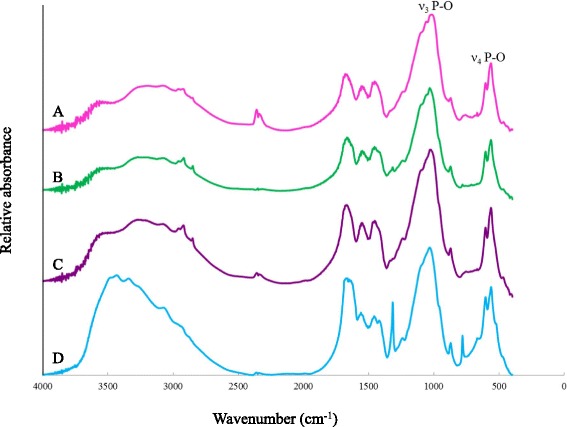
FT- IR absorption spectra of cementum: (A) CaP-F/Zn; (B) CaP-F; (C) CaP-Zn; (D) DDW. Note the spectra between 1300 cm^-1^ and 500 cm^-1^ showing greater resolution of PO_4_ absorption bands (at 1102 cm^-1^, 1065 cm^-1^, 1027 cm^-1^) in the spectra of materials scraped from cementum surface treated with CaP-F/Zn and CaP-F

In this study, FT- IR was applied to further explore the chemical features of treated cementum surfaces with coated layers. As shown in Fig. 2, F^-^ and Zn^2+^ doped apatite (CaP-F/Zn) or F^-^ alone doped apatite (CaP-F) treated cementum induced a slight rise in the resolution of the PO_4_ absorption band (ν3 P-O) at 1102, 1065, 1027 cm^-1^, which indicates an increase in crystal size and crystal perfection of the fluoride containing apatite on the treated cementum surfaces.

**Fig. 3:**
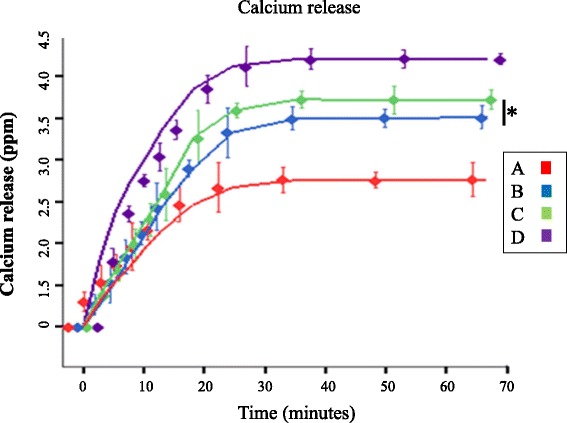
Comparative extent of Ca^2+^ ions released from cementum surfaces in acidic buffer: (A) CaP-F/Zn; (B) CaP-F; (C) CaP-Zn; (D) DDW. The amount of Ca^2+^ ions released in 60 min with acidic buffer challenge was the highest in the control group, and the lowest in CaP-F/Zn treated cementum group. *No significant difference was observed in the extent of Ca^2+^ ions released between cementum surfaces treated with CaP-F and CaP-Zn (*p* > 0.05, *n* = 3)

To prevent or halt the pathophysiological process of caries, the coating layers on the cementum would be preferred to possess the acid resistance properties. Thus in this study, dissolution experiments were carried out by measuring the released calcium from coated cementum surfaces during the acid attack using inductively coupled plasma (ICP) mass spectrometry. As shown in Fig. 3, the amount of Ca^2+^ ions released from cementum sections treated by CaP-F/Zn was the least after 1 h immersion in the acidic buffer (0.1 M KAc, pH 6, 37 °C). Compared with the DDW treated group, cementum surfaces treated by CaP-F or CaP-Zn released significant less Ca^2+^ ions (*p* < 0.01). There was no statistical significant difference between CaP-F and CaP-Zn with regards to the released calcium level (*p* > 0.05). To be more specific, the total amount of Ca^2+^ ions liberated from treated cementum surfaces during the acidic attack was determined using ICP (Table 3). The CaP-F/Zn treated surfaces had the lowest amount of liberated Ca^2+^ ions (2.11 ± 0.51 ppm) which was almost half of the Ca^2+^ ions liberated from DDW control group (3.84 ± 0.17 ppm). The cementum surfaces treated by CaP-F and CaP-Zn liberated similar amount of Ca^2+^ ions responding to the acidic attack with 3.01 ± 0.35 ppm and 3.26 ± 0.31 ppm respectively. It indicated that CaP-F/Zn provided the most durable cementum coating surfaces regarding the dissolution and acidic resistance over time.

**Table 3:** The amount of Ca^2+^ ions released from cementum surfaces after 66.7 min in acidic buffer

Groups	The amount of Ca^2+^ released after 66.7 min (ppm)
A	2.11 ± 0.51
B	3.01 ± 0.35^**^
C	3.26 ± 0.31^**^
D	3.84 ± 0.17^**##∆∆^

Group A, B, C or D treated with solutions CaP-F/Zn, CaP-F, CaP-Zn or DDW respectively. Compared with A, ^**^ *p* < 0.01; compared with B, ^##^ *p* < 0.01; compared with C, ^∆∆^ *p* < 0.01. *n* = 3

### Resistance against cariogenic bacterial colonization

**Fig. 4:**
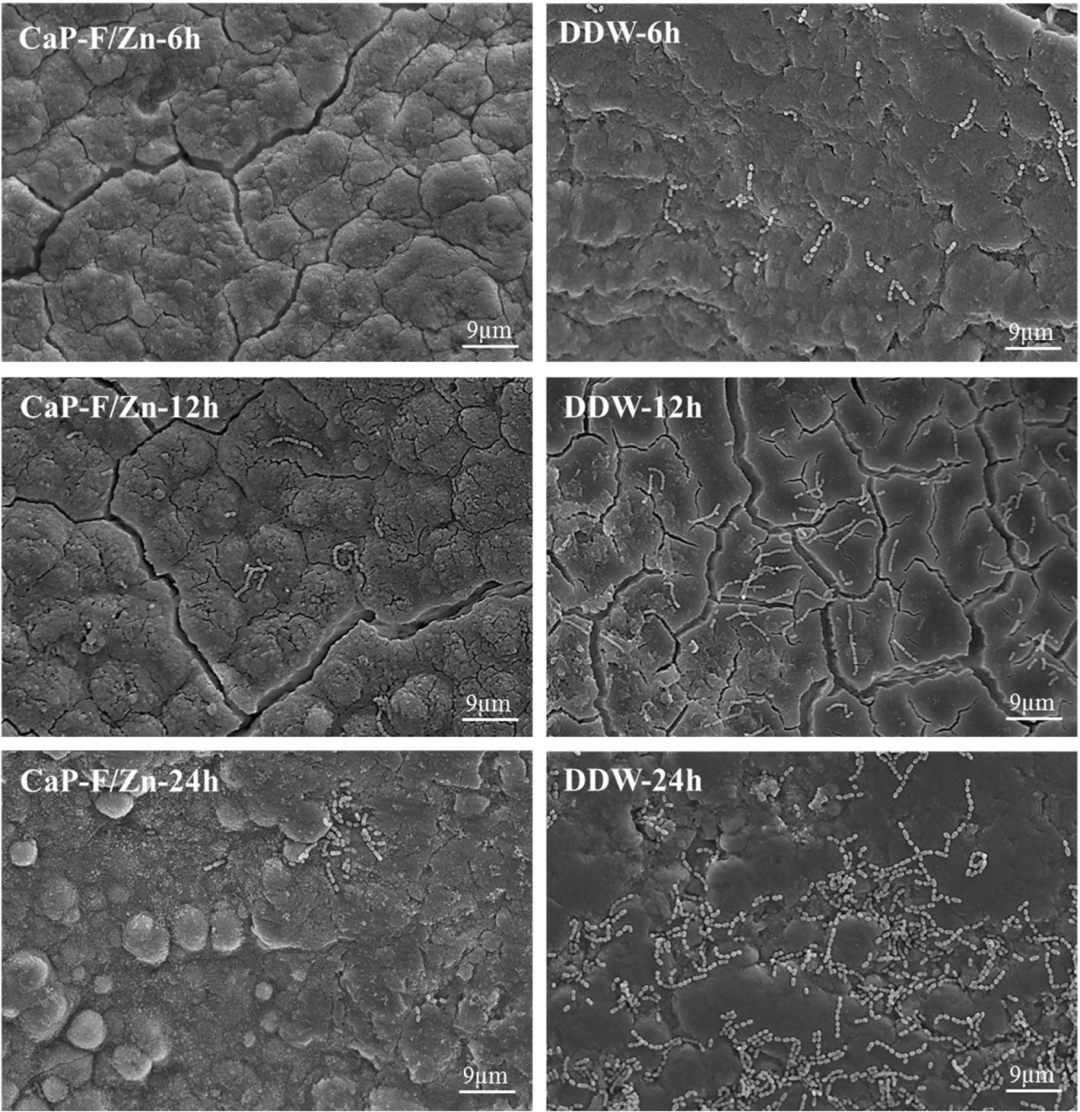
Representative SEM images of bacterial colonization on CaP-F/Zn or DDW treated cementum surfaces after *Streptococcus mutans* culturing 6, 12 and 24 h

**Fig. 5:**
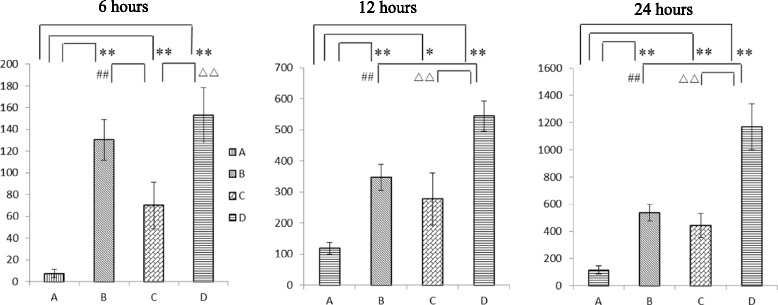
The mean number of *Streptococcus mutans* ATCC 25175 colonized on cementum surfaces were determined by average bacterial cells in ten randomly selected fields from each section (six sections in each group) and six SEM images for each section were captured. This experiment was independently repeated three times [41]. (A) CaP-F/Zn; (B) CaP-F; (C) CaP-Zn; (D) DDW (**p < 0.01; ^##^ *p* < 0.01; ^△△^ *p* < 0.01)

Colonization of material surfaces by cariogenic bacteria is a causal event which produces acid, initiates the tooth structure demineralisation, and eventually leads to caries cavitation. Hence, bacterial adherence and growth on M/A-CaPs treated surfaces were investigated using SEM to evaluate the resistance against cariogenic *S. mutans* colonization. As shown in Fig. 4, there was no significant increase of *S. mutans* population on CaP-F/Zn treated cementum from 6 to 24 h. In contrast, a dramatic proliferation of the bacterial population was observed on the cementum surfaces treated by DDW. Similar findings were also noticed on the CaP-F and CaP-Zn treated cementum surfaces (data not shown). To further quantify the bacterial colonization on various treated surfaces, the bacterial cell numbers were counted in defined regions of interest (ROIs) under SEM. As illustrated in Fig. 5, there was no significant difference between CaP-F and DDW treated groups at the 6 h and between CaP-F and CaP-Zn treated groups at 12 h or 24 h time points regarding the mean number of bacteria colonizing the cementum surfaces. However, the number of the bacteria colonized on CaP-F/Zn, CaP-F or CaP-Zn treated cementum surfaces was significantly less than DDW control group at 3 different time points. This may be explained by the fact that M/A-CaPs coating layers on the treated cementum surfaces release bacteriostatic and/or bactericidal ions such as Zn^2+^ and F^-^, thus inhibiting *S. mutans* growing on the M/A-CaPs treated cumentum surfaces. Among the three M/A-CaPs, the CaP-F/Zn treated cementum surfaces showed the best inhibiting effect against the colonization of *S. mutans* which indicated that the Zn^2+^ and F^-^ could have synergic effect.

## Discussion

Cementum is a calcified avascular mesenchymal tissue that covers the dentine and forms the outer layer of the anatomic root. There are two classes of cementum: Cellular cementum which contains cementocytes within the matrix and is mainly found in the apical area overlying the root; acellular cementum which is the one without any cells in its matrix and is located in the cervical and middle third of the root regions. Clinically, gingival recession and root surface exposure leads to the exposure of acellular cementum which predisposes the tooth to the development of root caries. In this study, only the mineralised ground acellular cementum was examined.

Treatment of cementum sections with three M/A-CaP solutions resulted in (a) deposition of a crystal precipitates coating layer on the cementum surface, (b) increasing resistance against acid dissolution, and (c) minimizing bacterial colonization. These effects were demonstrated to be dependent on the supplementation of F^-^ and/or Zn^2+^ in M/A-CaP solutions. In the presence of both F^-^ and Zn^2+^ in CaP-F/Zn, the treated cementum presented an improved performance in acid resistance and anti-bacterial property. Although the CaP-F coated the cementum with a similar smooth fine mineral precipitates layer to that of CaP-F/Zn, CaP-F performed poorly in acid resistance and bacterial inhibition. Notably, the CaP-Zn was not able to form a homogeneous fine layer of crystal precipitates whilst which could be seen on the cementum surfaces treated by CaP-F/Zn or CaP-F. It could be attributed to the larger crystalline formed in the presence of Zn^2+^leading to bulky crystal precipitates formation.

Under acidic condition with pH 5.5, the presence of F^-^ and Zn^2+^ in the traditional CaP solutions significantly affected the mineralisation status of cementum surfaces [19]. The carbonate hydroxyapatite on the cementum surface was partially dissolved to release Ca^2+^, Mg^2+^, HPO_4_^2-^, and CO_3_^2-^ ions. These irons subsequently combined with the Ca^2+^, Zn^2+^, HPO_4_^2-^, and F^-^ ions which were provided by the acid mineralising solutions, to form fluorapatite and/or Zn-doped apatite apatitic. Fluorapatite precipitates possessed lower solubility than the original cementum minerals which had a higher CO_3_^2-^ ion level and lower F^-^ ion level [28–30]. Similarly, the formed Zn-doped apatite also presented a reduced mineral solubility in acidic environment [22, 31]. One limitation of the current study was that the durability of the CaP-F/Zn precipitates on root surface was not determined. Future studies will be carried out to further investigate the stability and wearability of CaP-F/Zn precipitates under strong acidic environment and mechanical challenge.

The decrease in the prevalence and severity of dental caries has been attributed to the widely application of fluoride-containing dentifrices [32]. Although fluoride could affect the cariogenic ability of *Streptococcus mutans* by reducing carbohydrate metabolism and inhibiting certain enzymes activities, it has been demonstrated that fluoride cannot effectively kill oral streptococci in the biofilms [33–35]. Hence, other antimicrobial chemicals such as Zn^2+^ has been drawn a great deal of attention. Liberated Zn^2+^ ions can bind to essential metabolic enzymes in bacteria to achieve the bactericidal or bacteriostatic effects [36, 37]. Recently, McDevitt *et al* found that zinc bound to the manganese transport protein in human pathogen *Streptococcus pneumoniae* and inhibited the uptake of essential nutrient manganese for this bacterium [38]. Previous studies also demonstrated that zinc phosphate mineralised membranes effectively inhibited the *Actinobacillus actinomycetemcomitans* ATCC 29522 to colonize on root surface of the tooth [21]. In this study, we showed that CaP-F/Zn coating layer was more effective in minimizing bacterial growth and colonization compared with CaP-F or CaP-Zn. Liberated ions from CaP-F/Zn solution promoted the formation of F^-^ substitution and/or Zn^2+^ substitution apatite on the interface of coating layer and cementum underneath. Given the similarity of chemical components to human teeth hydroxyapatite, the M/A-CaP crystal precipitates coating layer processes promising biocompatibility. Furthermore, when bacteria attach on the cementum surface treated with CaP-F/Zn, acid produced by bacteria can dissolve the F^-^ substitution and/or Zn^2+^ substitution apatite leading to the release of F^-^ and Zn^2+^. The liberated F^-^ and Zn^2+^ ions could act synergistically in inhibition of bacterial colonization on the treated cementum surfaces. These results are in agreement with previous reports which have demonstrated that the combination of Zn^2+^ and F^-^ ions presented potential bactericidal effects [39, 40]. To further confirm the antimicrobial properties of CaP-F/Zn solution, the viable cell counts of adherent bacteria on cementum will be performed in future study.

## Conclusions

Clinically, different types of fluoride-containing agents such as varnish, solution or dentifrices have been applied to prevent and manage the root caries. The modified CaP mineralising solution supplemented with Zn^2+^and F^-^ has been demonstrated to be able to form an acid resistance shield with anti-cariogenic bacteria colonisation capacity in the current study. This new mineralisation solution could contribute to the development of a novel dentifrice to prevent and treat root cementum caries formation and progression.
